# Compound 3d Attenuates Metabolic Dysfunction-Associated Steatohepatitis via Peroxisome Proliferator-Activated Receptor Pathway Activation and Inhibition of Inflammatory and Apoptotic Signaling

**DOI:** 10.3390/metabo15050296

**Published:** 2025-04-29

**Authors:** Shouqing Zhang, Jiajia Yu, Sule Bai, Shuhan Li, Quanyuan Qiu, Xiangshun Kong, Cen Xiang, Zhen Liu, Peng Yu, Yuou Teng

**Affiliations:** China International Science and Technology Cooperation Base of Food Nutrition/Safety and Medicinal Chemistry, State Key Laboratory of Food Nutrition and Safety, Tianjin University of Science and Technology, Tianjin 300457, China; 96011955@mail.tust.edu.cn (S.Z.); yujiajia@mail.tust.edu.cn (J.Y.); bsl21914011@tust.edu.cn (S.B.); 23816895@mail.tust.edu.cn (S.L.); 15233102564@mail.tust.edu.cn (Q.Q.); qzz@mail.tust.edu.cn (X.K.); liuzhen5957@tust.edu.cn (Z.L.)

**Keywords:** elafibranor derivative, MASH, PPAR pathway activation, p38 MAPK signaling inhibition, gut microbiota modulation

## Abstract

**Objectives:** Metabolic dysfunction-associated steatohepatitis (MASH) lacks effective therapies. This study aimed to evaluate the therapeutic potential of compound **3d**, a novel elafibranor derivative, focusing on its dual mechanisms of PPAR pathway activation and p38 MAPK signaling inhibition. **Methods:** Integrated in vitro and in vivo approaches were employed. In vitro, free fatty acid (FFA)-induced lipid accumulation in L02 hepatocytes and lipopolysaccharides (LPSs)-stimulated inflammatory responses in RAW264.7 macrophages were used to evaluate lipid metabolism and anti-inflammatory effects. In vivo, a high-fat diet (HFD)-induced MASH model in C57BL/6 mice assessed serum biochemical parameters (triglycerides (TGs), total cholesterol (TC), low-density lipoprotein cholesterol (LDL-C), alanine aminotransferase (ALT), aspartate transaminase (AST), tumor necrosis factor-α (TNF-α), nitric oxide (NO), and interleukin-6 (IL-6)), liver histopathology (H&E, Oil Red O, Masson staining), and proteomic profiling. Gut microbiota composition was analyzed via 16S rRNA sequencing. Western blotting quantified PPAR isoforms (γ/δ), downstream targets (Acox1, EHHADH, Acaa1), and p38 MAPK pathway proteins (p-p38, caspase-8, Bcl-2). **Results:** In vitro, **3d** significantly reduced lipid accumulation (reduction in TG, *p* < 0.01) and inflammation (decrease in ALT activity, *p* < 0.05) in hepatocytes, while suppressing LPSs-induced TNF-α (63% reduction), NO (51% decrease), and IL-6 (48% reduction) in macrophages (*p* < 0.01). In vivo, **3d** (30 mg/kg) lowered serum TG (39% decrease), TC (32% reduction), LDL-C (45% decline), and TNF-α (57% reduction) in HFD-fed mice (*p* < 0.05 vs. model), normalized AST/ALT levels, and ameliorated hepatic steatosis, ballooning, and fibrosis. Proteomics demonstrated PPARγ/δ activation (2.3–3.1-fold upregulation of Acox1, EHHADH, Acaa1; *p* < 0.001) and p38 MAPK pathway inhibition (54% reduction in p-p38, 61% decrease in caspase-8; 1.8-fold increase in Bcl-2; *p* < 0.01). Gut microbiota analysis revealed enrichment of beneficial taxa (Lactobacillus: 2.7-fold increase; Bifidobacterium: 1.9-fold rise) and reduced pathogenic Proteobacteria (68% decrease, *p* < 0.05). **Conclusions:** Compound **3d** alleviates MASH via PPAR-mediated lipid metabolism enhancement and p38 MAPK-driven inflammation/apoptosis suppression, with additional gut microbiota modulation. These findings highlight **3d** as a multi-target therapeutic candidate for MASH.

## 1. Introduction

Metabolic dysfunction-associated steatohepatitis (MASH) is a complex progression of metabolic dysfunction-associated steatotic liver disease (MASLD), which, in the absence of timely intervention, may ultimately progress to cirrhosis and hepatocellular carcinoma [1]. As one of the leading causes of chronic liver injury, MASH represents a major global health concern. The pathogenesis of MASH is multifactorial, involving an intricate interplay of metabolic derangements, oxidative stress, and gut microbiota dysbiosis [2]. Current therapeutic strategies under development for MASH target diverse molecular pathways, including those implicated in lipid metabolism, inflammation, and fibrogenesis [3]. Despite extensive research in this field, the precise molecular mechanisms underlying MASH pathogenesis remain incompletely understood, necessitating further investigation to elucidate its etiology and identify novel therapeutic targets.

Peroxisome proliferator-activated receptors (PPARs) represent a subfamily of the nuclear hormone receptor superfamily [4]. These receptors have been shown not only to regulate lipid metabolism [5] but also to play critical roles in lipogenesis, hepatocyte differentiation, and hepatic fibrogenesis [6]. PPARs are classified into three major isoforms: PPARα, PPARγ, and PPARβ/δ [7]. Among these, PPARα governs hepatic lipolytic metabolism by inducing the expression of genes involved in mitochondrial and peroxisomal fatty acid oxidation [8]. PPARγ functions as a metabolic sensor, maintaining metabolic homeostasis and participating in the differentiation of adipocytes and macrophages [9]. Additionally, PPARγ has been demonstrated to suppress the production of pro-inflammatory cytokines in hepatic tissues, thereby exerting anti-inflammatory effects [10]. PPARβ/δ, meanwhile, plays diverse roles in energy metabolism and inflammatory regulation [11].

**Figure 1:**
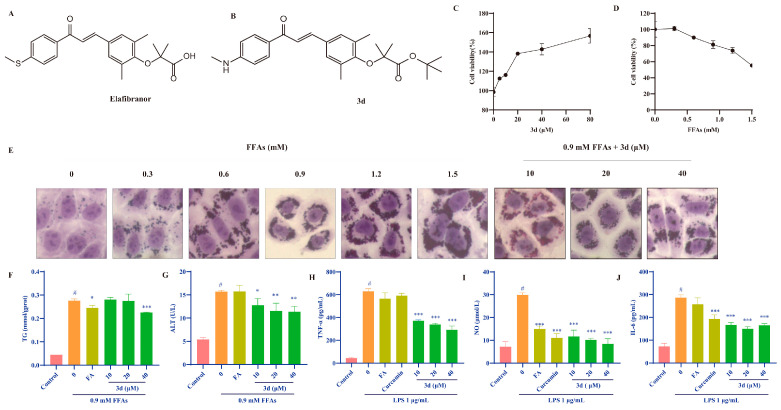
The effects of **3d** on models of lipid accumulation and inflammation. The structures of GFT505 (**A**) and **3d** (**B**). Compound **3d** facilitated the proliferation of L02 cells (**C**), whereas FFAs demonstrated the capacity to inhibit their growth (**D**). Cytomorphological observations indicated that **3d** was capable of alleviating FFA-induced lipid accumulation in L02 cells (**E**) and reduced TG (**F**) and ALT (**G**) levels. In the LPS-induced RAW264.7 cell inflammation model, **3d** was observed to significantly reduce the expression of inflammatory factors TNF-α (**H**), NO (**I**), and IL-6 (**J**). # *p* < 0.05 vs. Control; * *p* < 0.05, ** *p* < 0.01,*** *p* <0.001 vs. Model.

Elafibranor (GFT505, Figure 1A), a dual PPARα/δ agonist [12], failed to advance to Phase III trials due to insufficient efficacy and failure to meet predefined therapeutic endpoints [13]. Despite these limitations, elafibranor exhibited a favorable safety profile in clinical trials [14] and is currently approved for the treatment of primary biliary cholangitis [15]. Building on these findings, a series of elafibranor-derived compounds was designed and synthesized in a prior study, with compound **3d** (Figure 1B) identified as a promising therapeutic candidate for metabolic dysfunction-associated steatohepatitis (MASH) [16]. Compound **3d** was synthesized according to the procedure illustrated in Scheme S1, and its structural identity was unambiguously confirmed by comprehensive spectroscopic characterization. The ^1^H NMR spectrum (Figure S1) exhibited characteristic proton resonances corresponding to the proposed molecular framework, while the ^13^C NMR spectrum (Figure S2) displayed all expected carbon signals, both in full agreement with the anticipated structure. Purity assessment via high-performance liquid chromatography (HPLC, Figure S3) demonstrated an exceptional chemical purity of 99.19%, ensuring the reliability of subsequent experimental evaluations. 

In this study, we employed models of lipid accumulation and inflammatory cell response, as well as a high-fat diet (HFD)-induced MASH model, to investigate the therapeutic potential of compound **3d**. Proteomic analysis, 16S rRNA sequencing, and molecular biology approaches collectively demonstrated that compound **3d** alleviates MASH-associated lipid accumulation and inflammatory responses via PPAR pathway modulation. Furthermore, compound **3d** was shown to enhance the abundance of beneficial gut microbiota, suggesting a potential mechanism for its therapeutic effects in MASH.

## 2. Materials and Methods

### 2.1. In Vitro Lipid Accumulation Model

Free fatty acids (FFAs, OA:PA = 2:1) were used to induce the lipid model in L02 cells (Guangzhou Huatuo Biological Technology Co., Ltd., Guangzhou, China). The L02 cells (1 × 10^5^ cells/mL) were inoculated in 12-well plates. After 24 h, the cells were starved for 12 h and then treated with DMSO or FFAs for 24 h. Subsequently, the cells were exposed to fenofibric acid (100 μM) or **3d** (10, 20, 40 μM) for another 24 h.

The cells were collected and lysed using cell lysis solution; the supernatants were subjected to triglycerides (TGs) and alanine aminotransferase (ALT) kit assays. Moreover, following the completion of the treatment, the cells were fixed with 4% paraformaldehyde and stained with an Oil Red O, with the objective of observing the morphology under an inverted microscope.

### 2.2. In Vitro Inflammation Model

RAW264.7 cells were bought from Guangzhou Huatuo Biological Technology Co., Ltd. (Guangzhou, China). RAW264.7 cells (1 × 10^5^ cells/mL) were inoculated in 24-well plates. After 24 h, LPS (1 μg/mL) was added to induce cell inflammation. Subsequently, curcumin (100 μM), fenofibric acid (FA, 100 μM), or **3d** (10, 20, 40 80 μM) was added for 24 h. The cell supernatant was collected for analysis of inflammatory factors, including nitric oxide (NO), interleukin-6 (IL-6), and tumor necrosis factor (TNF-α).

### 2.3. Cell Apoptosis-Related Assays

L02 cells were inoculated in 6-well plates at 1 × 10^5^ cells/mL, and after completing the lipid-accumulating cell modeling treatment according to the method described in 2.1, the cells were collected and washed with 1× PBS buffer.

Reactive oxygen species (ROS) assay: ROS production was detected in the collected cells using a DCFH-DA fluorescent probe diluted in serum-free medium, and the stained cells were washed with 1× PBS buffer 3 times and then analyzed by flow cytometry.

Apoptosis test: Binding Buffer was used to resuspend the modeling-treated cells, cells were stained with Annexin V/PI, and apoptosis was determined using flow cytometry.

Mitochondrial membrane potential (MMP) test: After the cells were co-incubated with FFAs and drugs for 24 h, the remaining medium was discarded and the cells were washed with 1× PBS buffer before being treated with JC-1 probe dye. A laser confocal microscope was used to take photographs, which were quantitatively analyzed using ImageJ software (V. 1.52a, Wayne Rasband National Institutes of Health, Bethesda, MD, USA).

### 2.4. Animal Studies and Histological Analysis

All animal studies were conducted in accordance with national legislation and local guidelines at the Center for Laboratory Animals in Tianjin University of Science and Technology and approved by the Animal Ethics Committee of Tianjin University of Science and Technology (8 March 2022, Tianjin, China). The C57BL/6J male mice (6 weeks) were purchased from Henan Scribex Biotechnology Co, Ltd (Zhenzhou, China). Following a one-week period of acclimatization, the mice were fed a high-fat diet (HFD) comprising 60% fat (TP23300, Trophic Animal Feed High-Tech Co., Ltd., (Nantong, China)) and a low-fat control diet (TP23302, Trophic Animal Feed High-Tech Co., Ltd., (Nantong, China)) for 16 weeks.

After 16 weeks of continuous feeding on a high-fat diet, key indicators were tested to determine modeling success and drug administration was initiated. The mice were randomly divided into seven groups (n = 10) according to their body weight. The groups were as follows: control group (control diet, 0.5% CMC-Na), model group (HFD, 0.5% CMC-Na), positive control group (HFD, 100 mg/kg Fenofibrate), elafibranor group (HFD, 30 mg/kg), and **3d** group (HFD, 3, 10, 30 mg/kg). The experiment was conducted over a period of eight weeks.

After fasting for 12 h, the mice were anaesthetized and blood samples were collected, as were the mice liver tissues. The ileal contents were collected and immediately frozen in liquid nitrogen and then transferred to storage at −80 °C. The blood samples were subjected to centrifugation at 3000 rpm/min for 10 min to obtain supernatant serum, which was stored at −80 °C. The liver tissues were weighed, and a portion of the liver was preserved in 4% paraformaldehyde for subsequent pathological sectioning, including staining with H&E, Masson, and Oil Red O. The remaining samples were stored at −80 °C for testing of various biochemical indices.

### 2.5. Biochemical Analysis of Serum and Liver Tissue

The serum levels of TG, total cholesterol (TC), low-density lipoprotein cholesterol (LDL-C), glutathione aminotransferase (AST), ALT, and TNF-α were determined according to the instructions of the kit. The levels of glutathione (GSH) and superoxide dismutase (SOD) were measured using liver tissue homogenates. The kits were purchased from the Nanjing Jiancheng Institute of Biological Engineering (Nanjing, China).

### 2.6. The Sequencing of the Proteome and the 16S rDNA

Proteome sequencing of liver tissue samples was performed according to the specific requirements of the protocol by Beijing Qinglian Baiao Biotechnology Co., Ltd. (Beijing, China) [17]. Mouse fecal samples were collected and frozen in liquid nitrogen and stored at −80 °C.

Total DNA was extracted by QIAamp DNA Stool Mini Kit, homogenized by lysis buffer, digested by proteinase, and purified by a centrifugal column and then passed through NanoDrop and agarose gel electrophoresis for quality control. PCR amplification was performed with primers specific to the V3-V4 region of the bacterial 16S rRNA gene (341F: 5′-CCTAYGGGRBGCASCAG-3′; 806R: 5′-GGACTACNNGGGGTATCTAAT-3′), and the reaction system consisted of 2 × Taq PCR Master Mix, and the program was 95 °C pre-denaturation for 30. cycles (95 °C for 30 s, 55 °C for 30 s, 72 °C for 45 s) and extension at 72 °C for 10 min. After purification, bipartite sequencing was performed using the Illumina MiSeq PE300 platform. After QIIME2 quality control, the raw data were clustered into OTUs with 97% similarity, and the species were annotated based on the Silva database to analyze the Alpha/Beta diversity and differential flora.

### 2.7. Western Blotting Analysis

Proteins were extracted using RIPA lysate and a BCA kit for protein concentration, separated by SDS-PAGE gel electrophoresis, and transferred to PVDF membranes. The membranes were sealed with 5% skimmed milk and incubated at 4 °C overnight with primary antibodies, followed by co-incubation with the corresponding secondary antibodies. The protein bands were imaged using an infrared laser imager and quantitatively analyzed by ImageJ software. The antibodies used for Western blot were Acox1 (Abcam, ab184032), Acaa1 (Abcam, ab154091), EHHADH (Abcam, ab123490), PPARγ (Abcam, ab272718), PPARα (Abcam, ab314112), PPARδ (Abcam, ab178866), P38 (Abcam, ab31828), p-P38 (Abcam, ab195049), Bcl-2 (Abcam, ab182858), Caspase8 (Abcam, ab25901), anti-rabbit IgG (CST, 7074 S), and α-tubulin (Abcam, ab7291).

### 2.8. In Vitro Stability and Pharmacokinetic Studies of 3d

Elafibranor and **3d** (40 μM) were dissolved in DMEM medium, as well as in simulated gastric fluid (SGF) and simulated intestinal fluid (SIF), respectively, at 37 °C. The concentration of the compounds was determined within 24 h by HPLC. The HPLC conditions comprised an ACQUITY UPLC BEH C18 column maintained at 40 °C with a mobile phase consisting of 20% ultrapure water and 80% acetonitrile, with a flow rate of 1 mL/min at 360 nm.

The pharmacokinetics of **3d** in vivo were evaluated using male SD rats. Following a 12 h fast, the rats were administered **3d** (30 mg/kg) via gavage or tail vein injection, respectively, and blood samples were collected within 24 h for analysis of **3d** blood concentration via HPLC. The PK parameters were calculated using the DAS 3.0, and oral bioavailability (F) was obtained by the following formula.

F = AUCpo × Di.v/AUCi.v × Dpo × 100%(1)

### 2.9. Statistical Analysis

All data were expressed as mean ± standard deviation. The results were analyzed by one-way analysis of variance (ANOVA), and significant differences were determined by Duncan’s test using Graph Pad Prism 8.0 (San Diego, CA, USA). A *p*-value of less than 0.05 was considered statistically significant.

## 3. Results

### 3.1. D Reduced Lipid Accumulation and Inhibited Inflammatory Response In Vitro

The lipid accumulation cell model was established through the induction of L02 cells by FFAs. Fenofibric acid is approved by the FDA as a positive drug in combination with statins for the treatment of various types of hyperlipidemia [18]. Compound **3d** significantly increased cell survival, indicating that it is not toxic to these cells (Figure 1C). The FFA was observed to induce cell death in a dose-dependent manner, which suggests that lipotoxicity resulting from lipid accumulation may be a contributing factor in the observed cell death in L02 human hepatocytes (Figure 1D). The 0.9 mM FFA concentration was selected based on the observed state of the cells. Under this condition, **3d**-protected hepatocytes exhibited reduced susceptibility to lipotoxicity and diminished intracellular lipid accumulation (Figure 1E). Furthermore, **3d** treatment resulted in a notable reduction in intracellular TG levels (Figure 1F) and ALT activity (Figure 1G).

The impact of compound **3d** on inflammatory processes was evaluated using the LPS-induced RAW264.7 cell model, and curcumin, which has a significant anti-inflammatory effect, was used as the positive drug [19]. LPS resulted in a notable elevation in the expression of TNF-α (Figure 1H), NO (Figure 1I), and IL-6 (Figure 1J). Conversely, the levels of each inflammatory factor were significantly reduced after the addition of **3d**. The results at the cellular level indicate that compound **3d** has hepatoprotective and anti-inflammatory effects.

### 3.2. D Maintained Mitochondrial Function and Attenuated Apoptosis In Vitro

**Figure 2:**
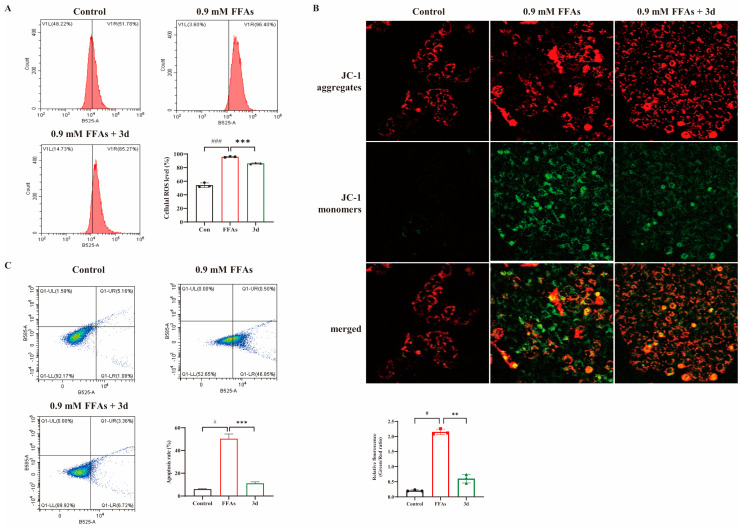
Effect of apoptosis in L02 cells. Compound **3d** was shown to inhibit FFA-induced intracellular ROS accumulation (**A**) stabilizing mitochondrial membrane potential (**B**) while inhibiting apoptosis (**C**). # *p* < 0.05, ### *p* < 0.001 vs. control group. ** *p* < 0.01, *** *p* < 0.001 vs. model. n = 3 per group.

To understand the mechanism by which FFAs reduce cell viability, the study first analyzed intracellular ROS production using flow cytometry, which was induced by FFAs with a significant increase in ROS levels and a significant decrease in ROS levels after **3d** administration (Figure 2A). It has been shown that mitochondrial damage is closely associated with hepatocyte apoptosis mediated by excess lipid accumulation [20]. Therefore, we evaluated the effect of **3d** on mitochondrial homeostasis under FFAs stimulation. It was shown that FFAs induced a severe depolarization of mitochondrial membrane potential (Figure 2B), which was partially restored after **3d** 20 μM treatment, and mitochondrial function was maintained. It further revealed that **3d** played a positive role in maintaining mitochondrial function. Mitochondrial dysfunction is an early stage of apoptosis [21], so the study measured apoptosis, which showed a significant increase after FFA treatment and a decrease to near-normal levels after **3d** treatment (Figure 2C). Excessive lipid accumulation can induce hepatocyte death by disrupting mitochondrial function [22]. Thus, **3d** could protect hepatocytes to reduce lipotoxicity-induced apoptosis.

### 3.3. D Effectively Inhibited HFD-Induced MASH Model in Mice

**Figure 3:**
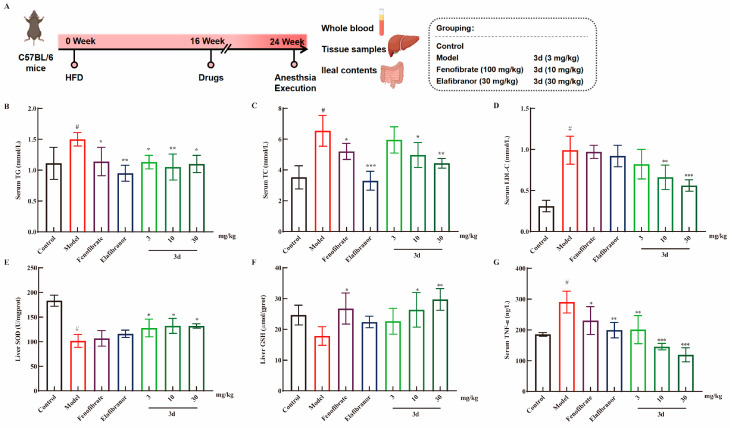
The effect of **3d** on MASH mice. The experiment was conducted in accordance with the established animal testing procedure (**A**). The levels of TG (**B**), TC (**C**), and LDL-C (**D**) were markedly elevated in the HFD-induced MASH model, whereas **3d** was observed to exert a significant inhibitory effect on their expression. The levels of SOD (**E**) and GSH (**F**) in the liver were found to be reduced as a consequence of the HFD, whereas their expression was observed to be enhanced by the **3d**. In addition, serum levels of TNF-α were observed to be elevated in MASH mice, whereas treatment with **3d** resulted in a significant reduction in its expression (**G**). # *p* < 0.05 vs. control group. * *p* < 0.05, ** *p* < 0.01, *** *p* < 0.001 vs. model. n = 6 per group.

The MASH model was induced using a high-fat diet (HFD) for a period of 16 weeks (Figure 3A). The most accumulated lipids in the liver during the development of fatty liver are TG and TC. The levels of TG and TC were found to be significantly elevated in mice fed HFDs. After the administration of fenofibrate, elafibranor, and **3d** treatment, serum TG levels decreased and returned to normal levels in all mouse groups (Figure 3B). Furthermore, a dose-dependent reduction in TC levels after **3d** treatment was observed in HFD mice (Figure 3C). TG and TC over-accumulation leads to an elevation of blood lipids [23]. LDL-C is the primary form of cholesterol transported within the body. Consequently, controlling LDL-C levels can assist in achieving lipid-lowering objectives [24]. It has been demonstrated that elevated LDL-C levels have been a contributing factor in the development of MASH [25]. In this study, an HFD diet was found to significantly elevate serum LDL-C levels in mice, and **3d** was observed to markedly diminish the serum levels of LDL-C in mice (Figure 3D). Furthermore, **3d** demonstrated antioxidant effects, counteracting the reduction in SOD (Figure 3E) and GSH (Figure 3F) content induced by an HFD diet. Moreover, an HFD diet resulted in elevated TNF-α levels in the serum of mice, which were reversed and returned to normal levels following **3d** treatment (Figure 3G).

**Figure 4:**
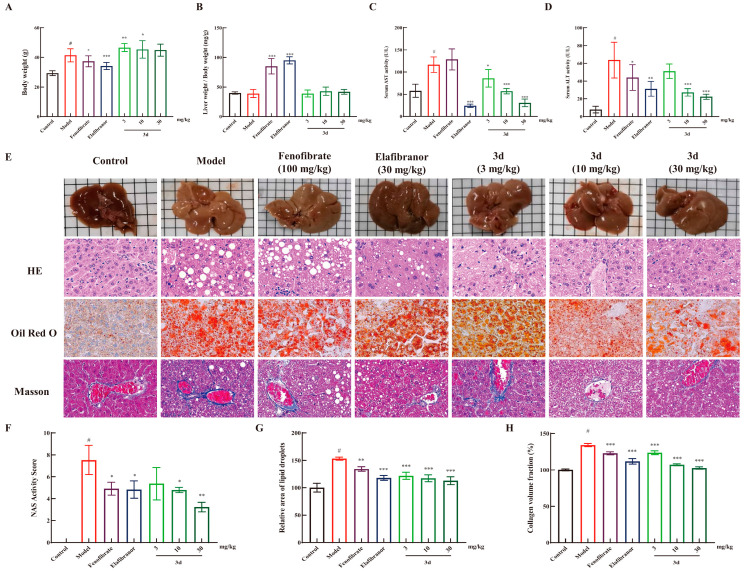
The protective effect of **3d** against liver injury in MASH mice. Compound **3d** did not affect the body weight (**A**) and liver weight (**B**) of the animals, and it significantly reduced the serum levels of AST (**C**) and ALT (**D**). Furthermore, **3d** ameliorated the pathological changes in the liver (**E**) and the corresponding quantitative analysis (**F**–**H**). # *p* < 0.05 vs. control group. * *p* < 0.05, ** *p* < 0.01, *** *p* < 0.001 vs. model. Scale bar, 50 μm. n = 6 per group.

Long-term HFD resulted in weight gain and concomitant increase in liver weight in mice (Figure 4A). The administration of fenofibrate and elafibranor resulted in a decrease in body weight, and **3d** had no significant effect on body weight. Liver weight/body weight is an indicator for evaluating drug toxicity. It is evident that fenofibrate and elafibranor exerted a substantial influence on the rise in liver weight/body weight, but not **3d** (Figure 4B). Concurrently, **3d** demonstrated a dose-dependent reduction in both AST and ALT activity (Figure 4C,D). The liver pathological changes of the mice were more visually compared between groups by staining the mouse liver sections (Figure 4E). Compared with the control group, the model group showed typical MASH features after long-term high-fat diet induction, with yellow coloration of the liver; HE section staining showed obvious steatosis and ballooning of hepatocytes in the liver; Oil Red section staining showed a large amount of fat in the liver, and fat was stained red; and Masson staining showed the appearance of fibrosis. The positive drug fenofibrate improved the steatosis and reduced lipid accumulation in the liver to some extent. Elafibranor was effective in reducing the degree of steatosis and reducing lipid accumulation better than fenofibrate but still could not bring them back to fully normal. After compound **3d** treatment, these histological changes were significantly alleviated, which could significantly reduce the steatosis and ballooning in the liver and bring it back to normal, and the lipid accumulation in mice was significantly reduced, and the liver fibrosis in mice was successfully reversed. NAS activity scores (Figure 4F) and quantitative analysis of Oil Red O (Figure 4G) and Masson staining (Figure 4H) presented the same results.

### 3.4. D Regulated Protein Expression in MASH Mice

**Figure 5:**
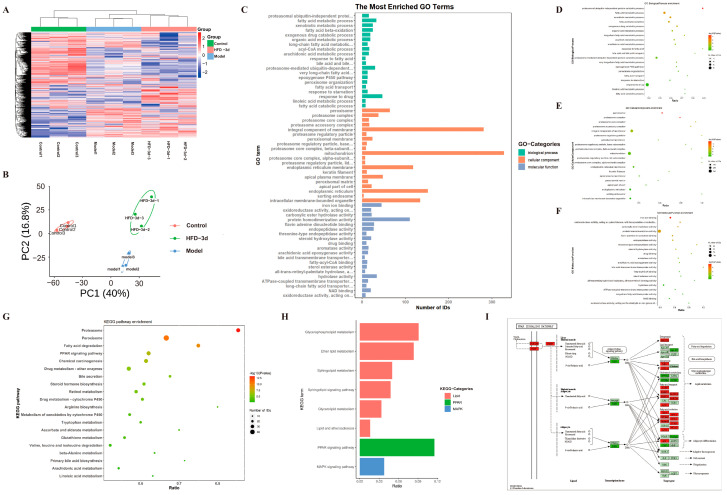
Proteomics analysis shows differential proteins in **3d**-treated HFD diet-fed mice. (**A**) The following heatmap depicts the differential protein expression in the control, model, and HFD-**3d** liver tissue. (**B**) A comparison of the control, model and HFD-**3d** liver tissue differential protein quantitative statistics, as well as a principal component analysis plot, is presented herewith. The histogram illustrates the distribution of GO enrichment (**C**), while the bubble plots depict the biological process (**D**), cellular component (**E**), and molecular function (**F**) for differential protein analysis in HFD-**3d** vs. model. Bubble plots of HFD-**3d** vs. the differential proteins were modeled in a KEGG enrichment analysis (**G**), with a particular focus on lipid metabolism, inflammation, and mitochondria-related pathways (**H**), as well as PPAR signaling pathways (**I**). n = 3 per group.

In order to systematically elucidate how **3d** treats MASH, the study performed proteomic analyses on HFD mice that received **3d** treatment. Cluster analysis heatmaps (Figure 5A) and PCA plots (Figure 5B) clearly divided the normal diet control group, the HFD model group, and the HFD diet group receiving **3d** treatment into three groups. The samples within the groups showed clear clustering within a certain range, similar protein expression patterns, small inter-sample differences, and stable reproducibility. Based on GO enrichment analysis, proteins associated with lipid metabolism, inflammatory response, and apoptosis were significantly changed after 3d treatment (Figure 5C–F). Meanwhile, its related protein pathways were similarly enriched in KEGG functional annotations (Figure 5G–H). Among them, the PPAR signaling pathway was closely related to MASH, and its downstream proteins were also changed (Figure 5I).

### 3.5. D Regulated PPAR Pathway to Inhibit Progression of MASH

**Figure 6:**
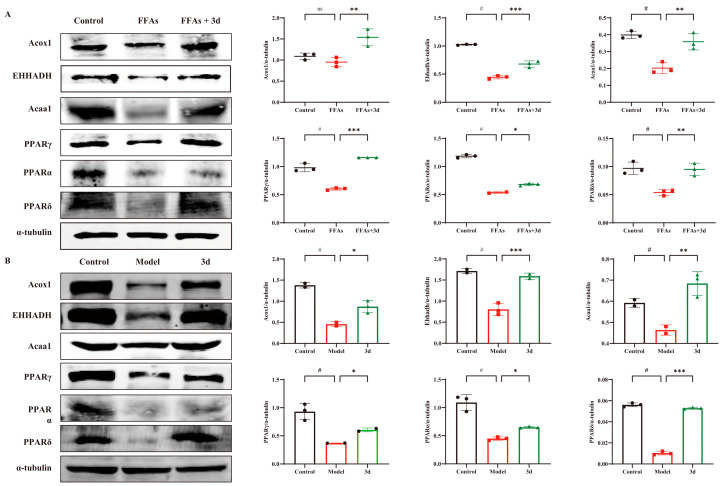
Effect of **3d** on PPAR-related proteins in cell and mice models. Expression of PPAR-associated proteins was markedly elevated in both cellular (**A**) and animal (**B**) models. Conversely, **3d** was observed to significantly reduce the expression of these proteins. # *p* < 0.05 vs. control group. * *p* < 0.05, ** *p* < 0.01, *** *p* < 0.001 vs. model. n = 3 per group.

The PPAR pathway performs crucial regulatory functions in fatty acid metabolism, inflammatory responses, and apoptosis. All of Acox1, EHHADH, and Acaa1 belong to the PPAR signaling pathway and are downstream target proteins of PPAR, reflecting the activation of PPAR. In FFA-stimulated L02 cells, PPAR-related proteins were significantly decreased, but they were significantly increased after exposure to **3d** (Figure 6A). The expression of PPAR α, γ, and δ was significantly reduced in liver tissues after dietary induction by HFD. The level of Acox1, EHHADH, and Acaa1 in the liver tissues of HFD-induced model group mice decreased with the decrease in PPAR expression. After **3d** administration, the decreasing trend of PPAR-related proteins was significantly reversed (Figure 6B). The above results suggest that **3d** has the potential to regulate lipids by activating the PPAR pathway.

### 3.6. D Inhibited Apoptosis via the P38 Inflammatory Pathway

**Figure 7:**
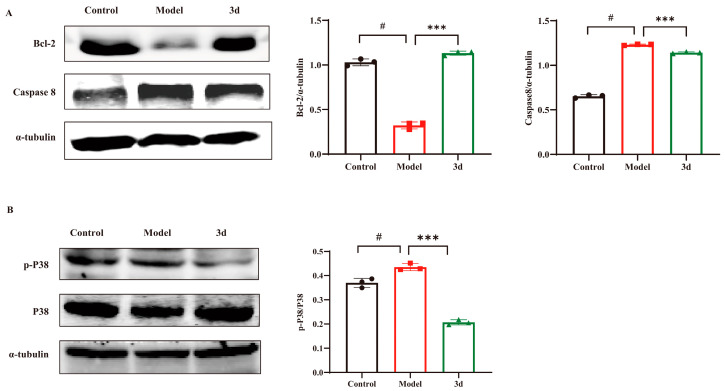
Effect of **3d** on proteins involved in inflammatory process and apoptosis in mice with HFD induced MASH. Compound **3d** markedly elevated Bcl-2 level and curtailed caspase-8 expression (**A**) while also markedly inhibiting P38 activation (**B**). # *p* < 0.05 vs. control group; *** *p* < 0.001 vs. model. n = 3 per group.

The aforementioned cellular assays demonstrated that **3d** was capable of stabilizing the mitochondrial membrane potential, thereby reducing apoptosis. Consequently, an examination was conducted to ascertain the expression of relevant apoptotic proteins. HFD resulted in activation of caspase 8 as well as down-regulation of the anti-apoptotic protein Bcl-2. In contrast, **3d** reversed the activation of caspase 8 and significantly up-regulated the expression of the anti-apoptotic protein Bcl-2 (Figure 7A), suggesting that **3d** restored apoptosis induced by HFD. An HFD diet significantly activated P38 protein phosphorylation in the liver, whereas the degree of P38 phosphorylation was significantly reduced after the administration of **3d**, which reduced the stimulation of various inflammatory factors and thus exerted a therapeutic effect on the inflammatory response during the disease process in MASH mice (Figure 7B). Therefore, the inhibition of apoptosis by attenuation of the inflammatory response through the P38 pathway may be one of the mechanisms of **3d** for MASH.

### 3.7. D Improves MASH by Modulating Gut Microbiota

**Figure 8:**
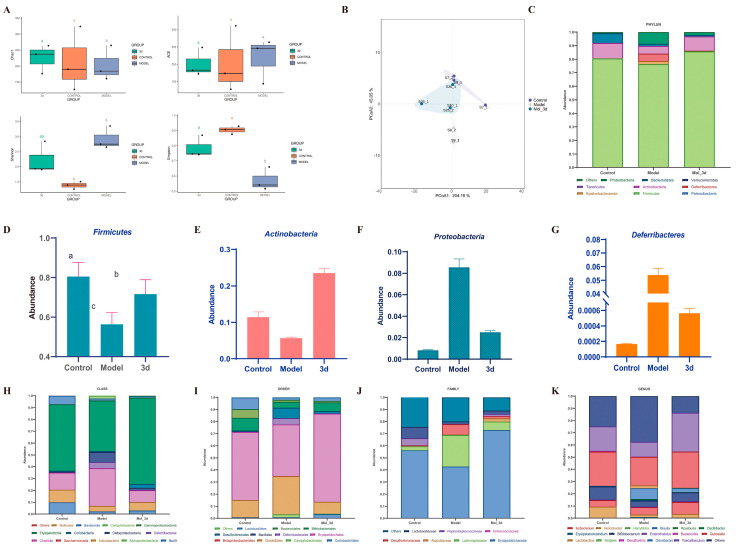
Effects of **3d** on intestinal flora. Alpha diversity (**A**), PCoA analysis (**B**), and species abundance at the phylum level (**C**) of **3d**. Abundance of *Firmicutes* (**D**), *Actinobacteria* (**E**), *Proteobacteria* (**F**), and *Deferribacteres* (**G**) of **3d**. Species abundance at the class level (**H**), order level (**I**), family level (**J**), and genus level (**K**) of **3d** treatment in MASH mice. Different letters mean *p* < 0.05. n  =  3 per group.

The human gut contains a highly complex and diverse array of microorganisms that have essential functions in host physiological processes [26]. There is mounting evidence that the gut microbiota may be a driver of disease progression in MASLD [27]. Targeting the gut microbiome is a novel therapeutic concept for combating the development of MASH. Microbial α-diversity (Figure 8A) showed no significant effect of **3d** on community richness effects of Chao1 and ACE indices. However, the Shannon index decreased and Simpson index increased in **3d** treated mice, and **3d** significantly reduced the gut microbial species diversity in MASH mice. The results of β-diversity showed (Figure 8B) that the gut microbial community after **3d** treatment was significantly separated from the model group, and there was a tendency for some of them to aggregate towards the blank group, indicating that the gut microbial community after **3d** treatment had a certain degree of similarity with that of the blank group. It was found that **3d** was effective in remodeling the structure of the gut microbial community of MASH mice. At the phylum level (Figure 8C), *Firmicutes* and *Actinobacteria* were the main dominant species, accounting for more than 80% of the overall abundance. The abundance of these two phyla was significantly increased after **3d** administration of the treatment (Figure 8D,E). The abundance of *Proteobacteria* (Figure 8F) and *Deferribacteres* (Figure 8G) was significantly reduced and returned to normal levels, and **3d** beneficially affected MASH mice at the levels of class, order, family, and genus (Figure 8H–K). Significant changes in *Lactobacillus* and *Bifidobacterium* occurred at the genus level after **3d** treatment, increasing the number of beneficial bacteria. Thus, **3d** could treat MASH by modulating the gut microbiota.

### 3.8. D Has Good Pharmacokinetic Properties

**Figure 9:**
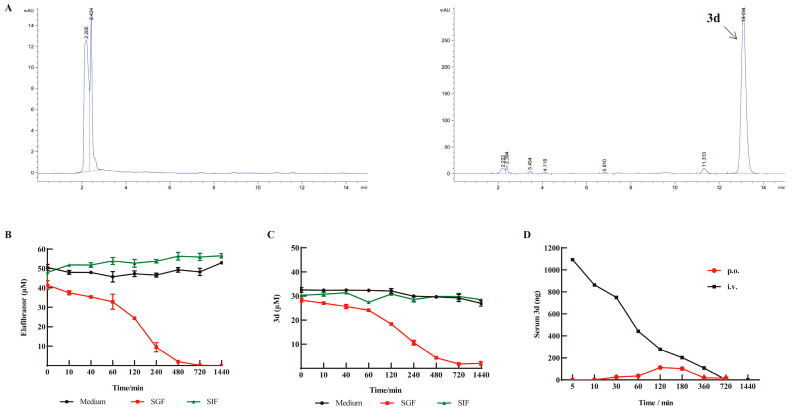
In vitro stability of **3d** and pharmacokinetics of **3d** in plasma. (**A**) HPLC chromatograms of blank plasma and **3d** in plasma. (**B**) Stability of elafibranor in vitro. (**C**) Stability of **3d** in vitro. (**D**) Blood concentration–time curve of **3d** in plasma. n  =  3. Pink line indicates HPLC integration range; Blue line.indicates peak of HPLC.

The stability of elafibranor and **3d** was evaluated in standard media, as well as in simulated gastric and intestinal fluids, in order to ascertain their suitability for use in such environments. The concentrations of elafibranor and **3d** in culture medium and intestinal fluid remained relatively constant, indicating that the compounds were stable under these conditions. However, in gastric fluid, the concentrations of the two compounds decreased significantly after 60 min (Figure 9B,C). The typical residence time of pharmaceuticals in the stomach is less than one hour, which allows for the oral administration of **3d**. Subsequently, pharmacokinetic testing was conducted for **3d** via both gavage and intravenous injection. As shown in Figure 9D, following gavage, the plasma concentration of **3d** was found to be markedly low, reaching a peak after two hours. In contrast, following intravenous injection, the plasma concentration of **3d** was observed to be significantly higher, demonstrating a gradual metabolic decline over time.

The pharmacokinetic parameters in rats administered orally for **3d** demonstrated that the peak concentration was reached at 2 h with a concentration of 113.14 ng/L. The half-life of **3d** was determined to be 3.4 h, with MRT_(0-t)_ of 4 h (Table 1). These findings suggest that **3d** is cleared from the body at a faster rate and has a shorter retention time. The oral bioavailability (**F**) of the **3d** was calculated to be 27.87%, which represents a relatively low level of bioavailability. Following intravenous administration, the half-life of **3d** is comparable to that observed following oral administration, which has been found to be 3 h. However, the AUC of **3d** was four times greater than that of the oral administration (Table 2). This indicates that **3d** intravenous administration has a rapid onset of action and high bioavailability but may also be associated with increased toxicity.

## 4. Discussion

MASH is defined as an abnormal accumulation of fat in the liver, accompanied by inflammation and hepatocellular damage. The principal pharmacological agents currently under investigation for the treatment of MASH are PPAR agonists, FXR agonists, and ACC inhibitors [28]. The successful launch of saroglitazar, a drug that acts on PPAR, has also demonstrated the feasibility of treatment on this target. Elafibranor, a selective PPAR α/δ agonist, has been demonstrated to exert a protective effect against hepatic steatosis, liver inflammation, and liver fibrosis [29]. However, in clinical phase III trials, it did not prevent the progression of tissue stasis and fibrosis due to an inadequate level of efficacy. Nevertheless, it is encouraging to note that elafibranor has been approved for the treatment of primary biliary cholangitis in 2024 [15], which indicates that it possesses clear pharmacological properties [30]. A series of elafibranor derivatives were previously designed and synthesized in our laboratory. Following experimental screening, it was found that compound **3d** exhibited superior activity and low toxicity to elafibranor and was potentially useful for MASH [16]. In the present study, a range of cellular and animal models were employed to provide further validation of the effect of **3d** on MASH, in addition to analyzing its potential mechanism.

The complex pathogenesis of MASH results in the simultaneous occurrence of multiple factors, including abnormal lipid metabolism, lipotoxicity, oxidative stress, and mitochondrial dysfunction, which act on the liver to trigger inflammatory responses and fibrotic lesions, thereby accelerating the deterioration of MASH. The PPAR pathway plays a pivotal role in the regulation of lipid metabolism and inflammatory responses in MASH. In addition, the activation of the PPAR pathway has been demonstrated to impede lipid accumulation and inflammation [4]. Elafibranor is a PPARα/δ dual agonist [29], and **3d** also regulates related proteins in the PPAR pathway. The administration of **3d** was observed to significantly enhance the expression of PPAR α, γ, and δ, with a notable increase in the latter two isoforms. In addition, **3d** could elevate the expression of PPAR downstream proteins Acox1, EHHADH, and Acaa1. Acox1 is an important protein involved in the metabolic pathway of fatty acid oxidation [31]. It acts as a key rate-limiting enzyme catalyzing peroxisomal β-oxidation and mediates oxidative stress leading to mitochondrial damage and reduced mitochondrial β-oxidation capacity [32]. EHHADH, involved in the fatty acid β-oxidation metabolic pathway, is responsible for hydration and dehydrogenation reactions and is one of the major oxidative metabolic pathways in the body for fats, which contributes to the maintenance of lipid metabolism homeostasis. The down-regulation of EHHADH expression has been reported to decrease the rate of very low-density lipoprotein (VLDL) transport, and accumulation of high levels of VLDL increases the risk of atherosclerosis and cardiovascular disease [33]. Acaa1, an important enzyme protein involved in fatty acid metabolism, is involved in the metabolism of acetyl-CoA in the fatty acid β-oxidation metabolism pathway. The Acaa1 protein can influence the development and progression of MASH by regulating lipid metabolic pathways. Furthermore, Acaa1 is involved in the regulation of oxidative stress and inflammation and influences the pathological process of MASH by affecting the balance of redox reactions and curbing liver injury and fibrosis [34]. Our findings demonstrated that **3d** instigates the activation of PPAR and its downstream pathway, which in turn led to the inhibition of lipid accumulation. It is of great significance to note that inflammation represents a pivotal characteristic response in MASH. The present study demonstrated that **3d** significantly reduced the intracellular inflammatory response triggered by LPS in RAW264.7 cells. This reduction was attributed to the attenuation of the production of NO, IL-6, and TNF-α, which are inflammatory mediators. The anti-inflammatory mechanism of **3d** may be associated with the p38 pathway. p38 is a class of serine/threonine kinases whose activation induces the NF-κB signaling pathway and stimulates the expression of its downstream inflammatory factors, including IL-1β, IL-6, and TNF-α. p38 inhibitors have a beneficial effect on steatohepatitis in mice fed a high-fat, high-cholesterol diet [35]. Therefore, it is hypothesized that p38 may represent a viable pathway or target for MASH therapy. A recent study demonstrated that elafibranor inhibited the expression of adipogenesis-related proteins in differentiated 3T3L-1 cells, including ERK, JNK, p38, and so on, proving its effectiveness in suppressing intracellular lipid accumulation [36], which was consistent with our results.

The accumulation of excess lipids has been demonstrated to induce mitochondrial dysfunction, which in turn can lead to the process of apoptosis. It has been reported that the inhibition of apoptosis can effectively control tissue scarring and curb hepatic fibrosis. Furthermore, FFA has been shown to cause a significant reduction in ROS levels, thereby activating oxidative stress [37]. However, **3d** has been shown to effectively protect L02 cell activity and maintain mitochondrial membrane potential homeostasis. Mitochondrial dysfunction represents an initial stage of apoptosis. The characteristics of mitochondria-induced apoptosis include a collapse of the mitochondrial membrane potential, ATP deficiency, the release of pro-apoptotic factors, and the activation of the caspase family. The activation of the cysteine asparaginase family, which is induced by exogenous FFAs, results in the release of pro-apoptotic factors from the mitochondria into the cytoplasm/nucleus, accompanied by a corresponding down-regulation of anti-apoptotic proteins [20]. In order to validate the **3d** for the treatment of MASH, proteomics was introduced as a means of analyzing the differential proteins. The findings revealed that **3d** exhibited superior functions pertaining to the regulation of lipid metabolism, hepatoprotection, anti-inflammation, and maintenance of mitochondrial homeostasis.

The composition and diversity of the intestinal flora influences host metabolic functions, including fat metabolism [38] and inflammatory responses [39]. Dysregulated intestinal flora may lead to liver fat deposition and inflammation, thereby exacerbating the pathologic process of MASH. *Firmicutes* are involved in bile acid metabolism, and the uncoupling enzyme bile salt hydrolase is synthesized by *Firmicutes* during bile acid metabolism [40]. The process of bile acid metabolism plays a key role in many physiological processes, not only contributing to fat digestion and absorption but also influencing the composition of the intestinal flora and energy homeostasis, and the interaction between bile acids and the intestinal flora is bidirectional [41]. The abundance of *Firmicutes* significantly increased to normal levels after the administration of **3d** treatment, which is favorable for regulating the bile acid cycle, maintaining the diversity of symbiotic bacterial communities and keeping their balanced growth. Decreased populations of *Actinobacteria* are strongly associated with obesity, diabetes, and immune response. *Actinobacteria* are one of the four major phyla of the intestinal flora [42] and promote tight junction function and maintenance of intestinal integrity [43]. The **3d** treatment significantly increased the abundance of *Actinobacteria phylum*, which is conducive to a reduction in inflammation in the intestinal tract, enhancement of the intestinal mucosal barrier, and protection of intestinal microecological stability.

Compound **3d** was shown to be a potential therapeutic agent for the treatment of MASH in our previous and present study experiments; therefore, its stability and pharmacokinetic parameters were investigated. Compound **3d** demonstrated superior stability in culture fluid, with the greatest stability observed in culture fluid and simulated intestinal fluid and less pronounced stability in simulated gastric fluid. The oral bioavailability of **3d** is comparable to elafibranor at around 30% [12]. The oral **3d** displays a favorable in vivo metabolic profile, exhibiting rapid absorption within the body. Moreover, we found elafibranor elevated liver weight/body weight in mice. It suggests some toxicity of elafibranor, which is consistent with the reported side effects of abdominal pain, diarrhea, nausea and vomiting with continuous administration of elafibranor [14,15], leading to weight loss in mice. Nevertheless, **3d** does not exhibit the same degree of toxicity, and on the whole, **3d** has the potential to be superior to elafibranor.

**Figure 10:**
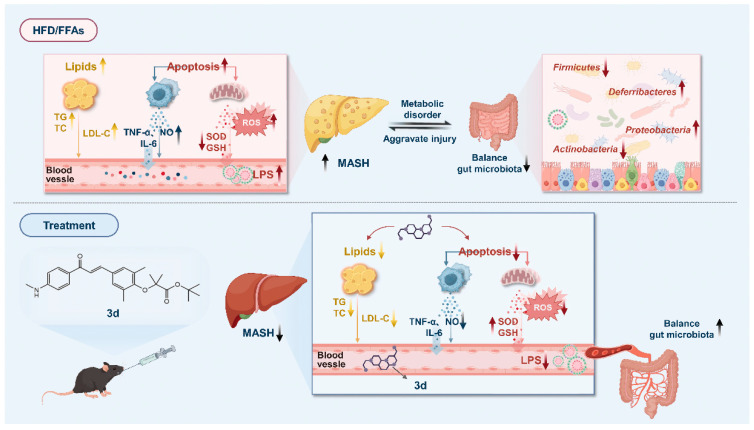
A proposed schematic model of the possible underlying molecular mechanisms associated with the therapeutic effect of **3d** against MASH.

In conclusion, the present study identifies **3d** as a potential therapeutic drug molecule for the treatment of MASH, with a mechanism involving the regulation of lipid metabolism, inflammatory response, and apoptosis via the PPAR pathway (Figure 10).

## 5. Conclusions

The elafibranor-derived compound **3d** demonstrates therapeutic efficacy against MASH. In vitro, **3d** reduced lipid accumulation (TG/ALT) and inflammation (TNF-α/NO/IL-6) in hepatocytes and macrophages. In HFD-fed mice, 3d lowered serum TG, TC, LDL-C, and TNF-α, normalized AST/ALT, and improved hepatic histopathology. Mechanistically, **3d** activated PPARγ/δ signaling (up-regulating Acox1, EHHADH, Acaa1), suppressed p38 MAPK phosphorylation and caspase-8, and enhanced Bcl-2. Gut microbiota modulation via *Lactobacillus/Bifidobacterium* enrichment further supported efficacy. These findings position 3d as a dual-target MASH therapeutic candidate through PPAR activation and p38 MAPK inhibition, underpinning its clinical potential.

## Figures and Tables

**Table 1:** Pharmacokinetic parameters of **3d** administered by gavage.

Parameters	Unit	Value	Parameters	Unit	Value
AUC_(0-t)_	ng/L*min	30,236	Zeta	1/min	0.003
AUC_(0-∞)_	ng/L*min	34,864	Zeta		134
AUMC_(0-t)_		7,351,242	C_z_	ng/L	15
AUMC_(0-∞)_		12,023,223	T_1/2_	min	204
MRT_(0-t)_	min	243	T_max_	min	120
MRT_(0-∞)_	min	344	V_Z/F_	L/kg	177,980
VRT_(0-t)_	min^2^	31,533	CL_Z/F_	L/min/kg	602
VRT_(0-∞)_	min^2^	107,286	C_max_	ng/L	113

**Table 2:** Pharmacokinetic parameters of **3d** intravenous administration.

Parameters	Unit	Value	Parameters	Unit	Value
AUC_(0-t)_	ng/L*min	108,480	Zeta	1/min	0.004
AUC_(0-∞)_	ng/L*min	136,923	Zeta		123
AUMC_(0-t)_		11,816,712	C_z_	ng/L	107
AUMC_(0-∞)_		29,781,860	T_1/2_	min	182
MRT_(0-t)_	min	108	T_max_	min	5
MRT_(0-∞)_	min	217	Vz	L/kg	40,444
VRT_(0-t)_	min^2^	10,194	CL_Z_	L/min/kg	153
VRT_(0-∞)_	min^2^	66,630	C_max_	ng/L	1092

## Data Availability

Data available on request from the authors.

## Supplementary Materials

The following supporting information can be downloaded at: https://www.mdpi.com/article/10.3390/metabo15050296/s1, Scheme S1: Synthesis method of **3d**; Figure S1: 1H-NMR spectrum of compound **3d**; Figure S2: 13C-NMR spectrum of compound **3d**; Figure S3: HPLC spectrum of compound **3d**.

## Abbreviations

The following abbreviations are used in this manuscript: PPARs: peroxisome proliferator-activated receptors; MASH, metabolic dysfunction-associated steatohepatitis; FFA, free fatty acid; TC, total cholesterol; ALT, alanine aminotransferase; LPS, lipopolysaccharide; TNF-α, tumor necrosis factor-α; IL, interleukin; HFD, high-fat diet; TG, triglyceride; LDL-C, low-density lipoprotein cholesterol; AST, aspartate aminotransferase; ACOX1, acyl-CoA oxidase 1; EHHADH, peroxisomal L-bifunctional enzyme; ACAA1, acetyl-CoA acyltransferase 1; MASLD, metabolic dysfunction-associated steatotic liver disease; OA, oleic acid; PA, palmitic acid; DMSO, dimethyl sulfoxide; FA, fenofibric acid; NO, nitric oxide; ROS, reactive oxygen species; MMP, mitochondrial membrane potential; CMC-Na, carboxymethylcellulose sodium; GSH, glutathione; SOD, superoxide dismutase; SDS-PAGE, sodium dodecyl sulfate polyacrylamide gel electrophoresis; PVDF, polyvinylidene difluoride; SGF, simulated gastric fluid; SIF, simulated intestinal fluid; HPLC, high-performance liquid chromatography; HE, hematoxylin-eosin staining; PCA, principal component analysis; GO, gene ontology; KEGG, Kyoto Encyclopedia of Genes and Genomes; ACE, angiotensin-converting enzyme; FXR, farnesoid X receptor; ACC, acetyl-CoA carboxylase; NF-κβ, nuclear transcription factor-kappa B; VLDL, very low-density lipoprotein; MAPK, mitogen-activated protein kinase; ATP, adenosine triphosphate; SCFAs, short-chain fatty acids.
