# Supplementary material for: Compound 3d Attenuates Metabolic Dysfunction-Associated Steatohepatitis via Peroxisome Proliferator-Activated Receptor Pathway Activation and Inhibition of Inflammatory and Apoptotic Signaling
Source: Metabolites. 2025 Apr 29;15(5):296. doi: 10.3390/metabo15050296 (PMC12113347; doi:10.3390/metabo15050296)
Supplement: Supplementary file 1 [file metabolites-15-00296-s001.zip › metabolites-3588595-supplementary.pdf]

Compound 3d Attenuates Metabolic Dysfunction-Associated Steatohepatitis via PPAR Pathway Activation and Inhibition of Inflammatory and Apoptotic Signaling

Shouqing Zhang #, Jiajia Yu #, Sule Bai, Shuhan Li, Quanyuan Qiu, Xiangshun Kong, Cen Xiang, Zhen Liu, Peng Yu * and Yuou Teng *

China International Science and Technology Cooperation Base of Food Nutrition/Safety and Medicinal Chemistry,

State Key Laboratory of Food Nutrition and Safety, Tianjin University of Science and Technology, Tianjin, 300457, China.

* Correspondence: yupeng@tust.edu.cn (P.Y.); tyo201485@tust.edu.cn (Y.T.)

# These authors contributed equally to this work.

CONTENT

Scheme S1. Synthesis method of 3d 2
Synthesis method of 3d 3
Figure S1. ^{1}H-NMR spectrum of compound 3d 4
Figure S2. ^{13}C-NMR spectrum of compound 3d 5
Figure S3. HPLC spectrum of compound 3d 6

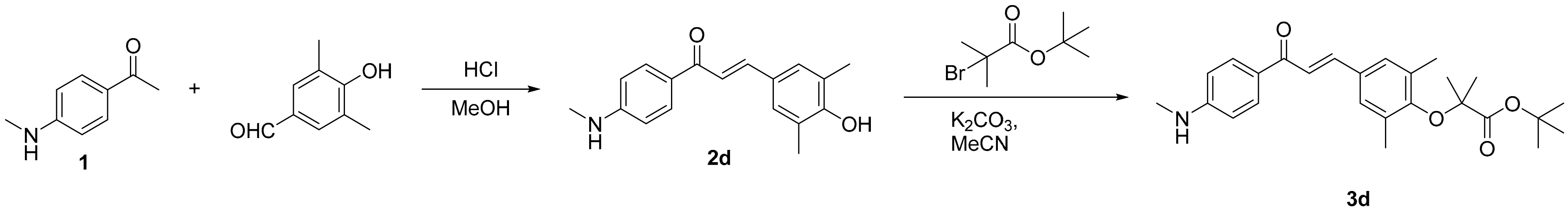

Scheme S1. Synthesis method of 3d.

Synthesis method of 3d

(*E*)-3-(4-hydroxy-3,5-dimethylphenyl)-1-(4-(methylamino)phenyl)prop-2-en-1-one(2d). The starting material 1-(4-(methylamino)phenyl)ethan-1-one (2.0 g, 13.41 mmol) was added to the solution of 4-hydroxy-3,5-dimethylbenzaldehyde (2.01 g, 13.41 mmol) in HCl/MeOH (4M, 2.5 equiv.), then 3.32 g of **2d** was obtained with 88% of yield.

(*E*)-2-(2,6-dimethyl-4-(3-(4-(methylamino)phenyl)-3-oxoprop-1-en-1-yl)phenoxy)-2-methylpropanoate(3d). The starting material **2d** (3.0 g, 10.66 mmol) was added to the solution of *tert*-butyl 2-bromo-2-methylpropanoate (1.98 mL, 10.63 mmol) and K_2CO_3 (2.2 g, 15.94 mmol) in MeCN, then 3.25 g of **3d** was obtained with 72% of yield. The purity of **3d** was 99.2%.^{1}H NMR (400 MHz, $CDCl_3$) δ 7.96 (d, *J* = 8.8 Hz, 2H), 7.67 (d, *J* = 15.6 Hz, 1H), 7.45 (d, *J* = 15.6 Hz, 1H), 7.26 (s, 2H), 6.60 (d, *J* = 8.8 Hz, 2H), 4.34 (s, 1H), 2.91 (s, 3H), 2.26 (s, 6H), 1.51 (s, 9H), 1.45 (s, 6H). ^{13}C NMR (100 MHz, $CDCl_3$) δ 187.9, 173.2 , 155.2, 153.2, 142.4, 133.5, 131.0, 128.7, 127.2, 121.1, 111.2, 81.6, 30.1, 27. 9, 27.8, 25.6, 18.3.HRMS (+ESI-TOF) *m*/*z*: $[M + Na]^+$ Calcd. for $C_{26}H_{33}NO_4$ 446.2302; Found 446.2302.

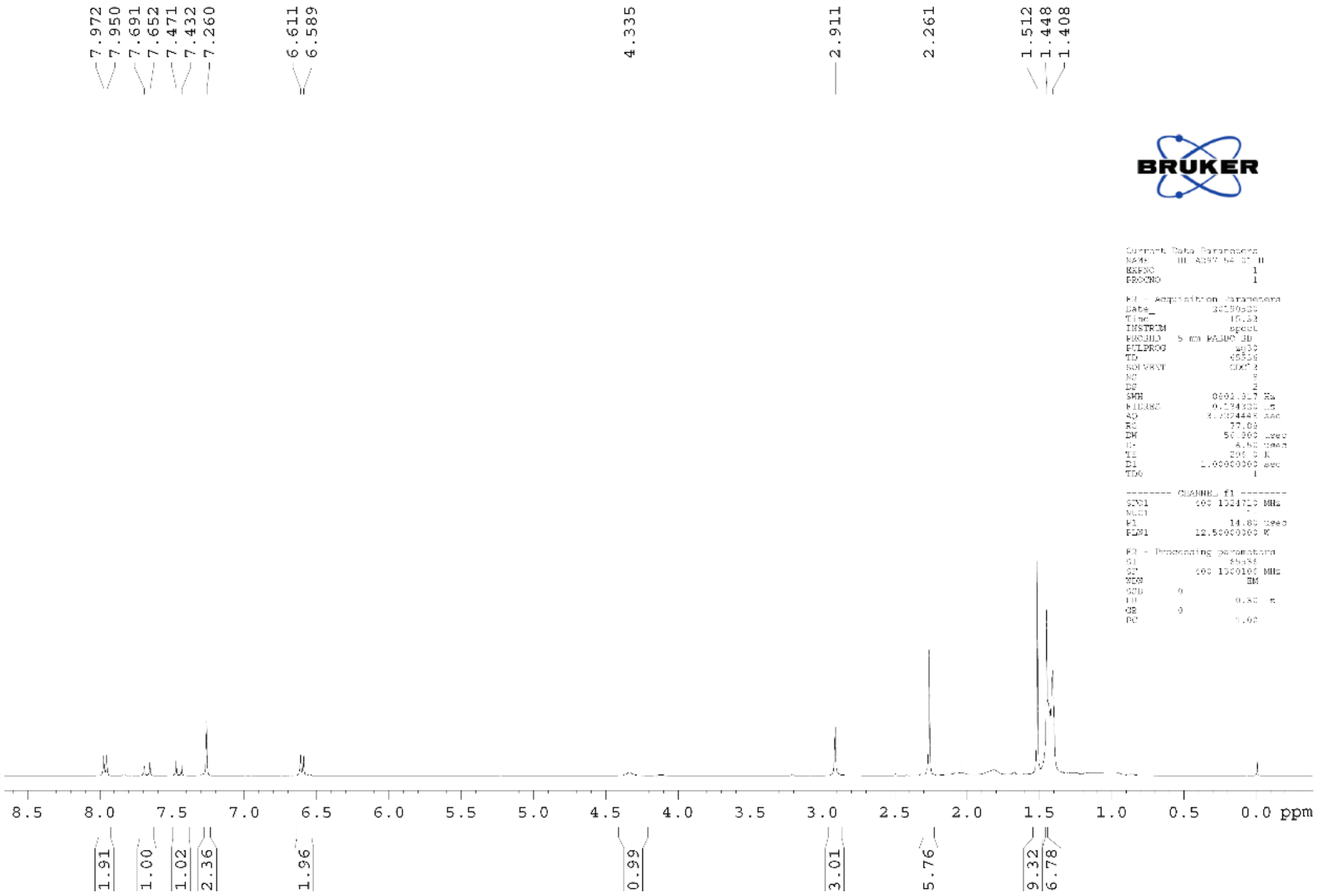

Figure S1. ^{1}H-NMR spectrum of compound 3d.

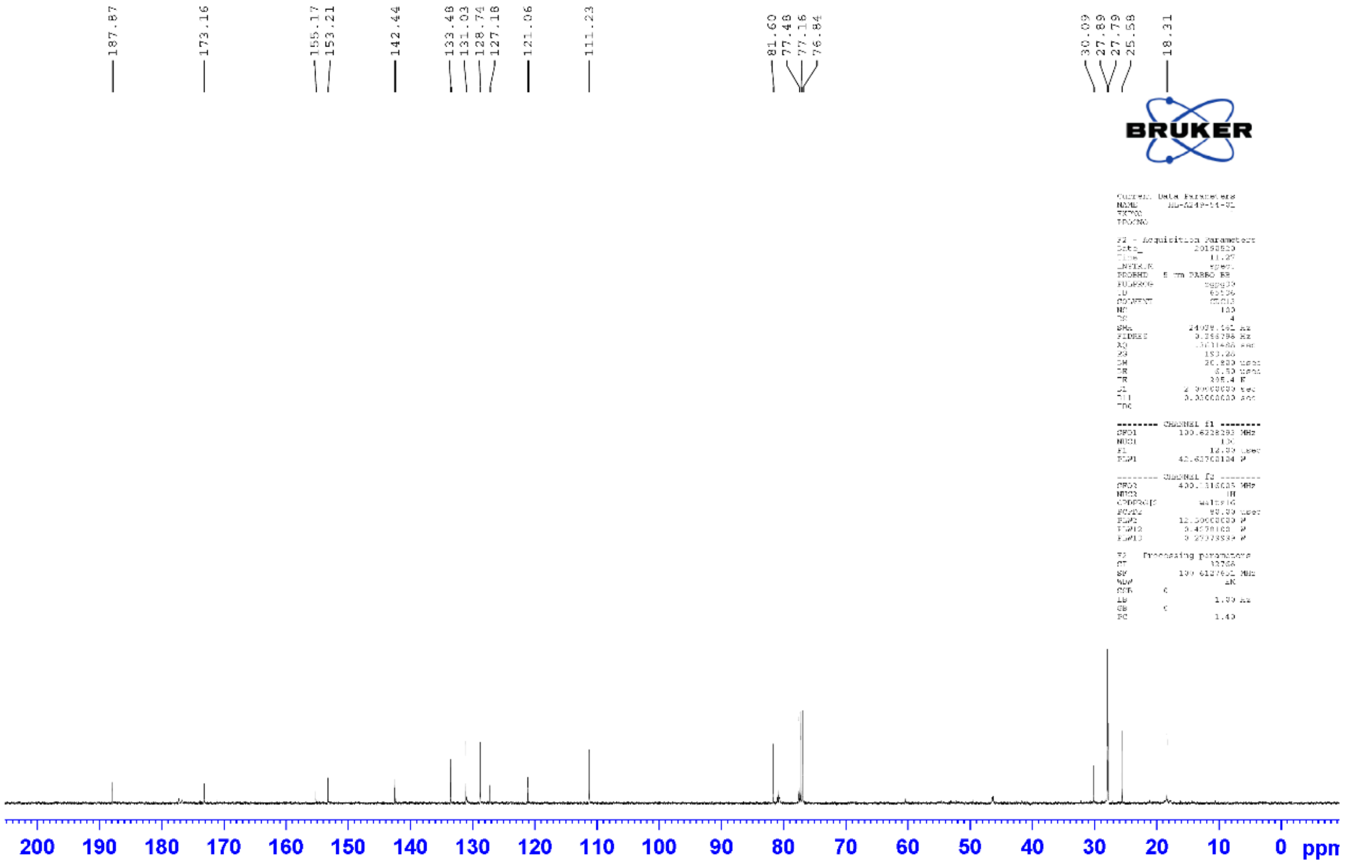

Figure S2. ^{13}C-NMR spectrum of compound 3d.

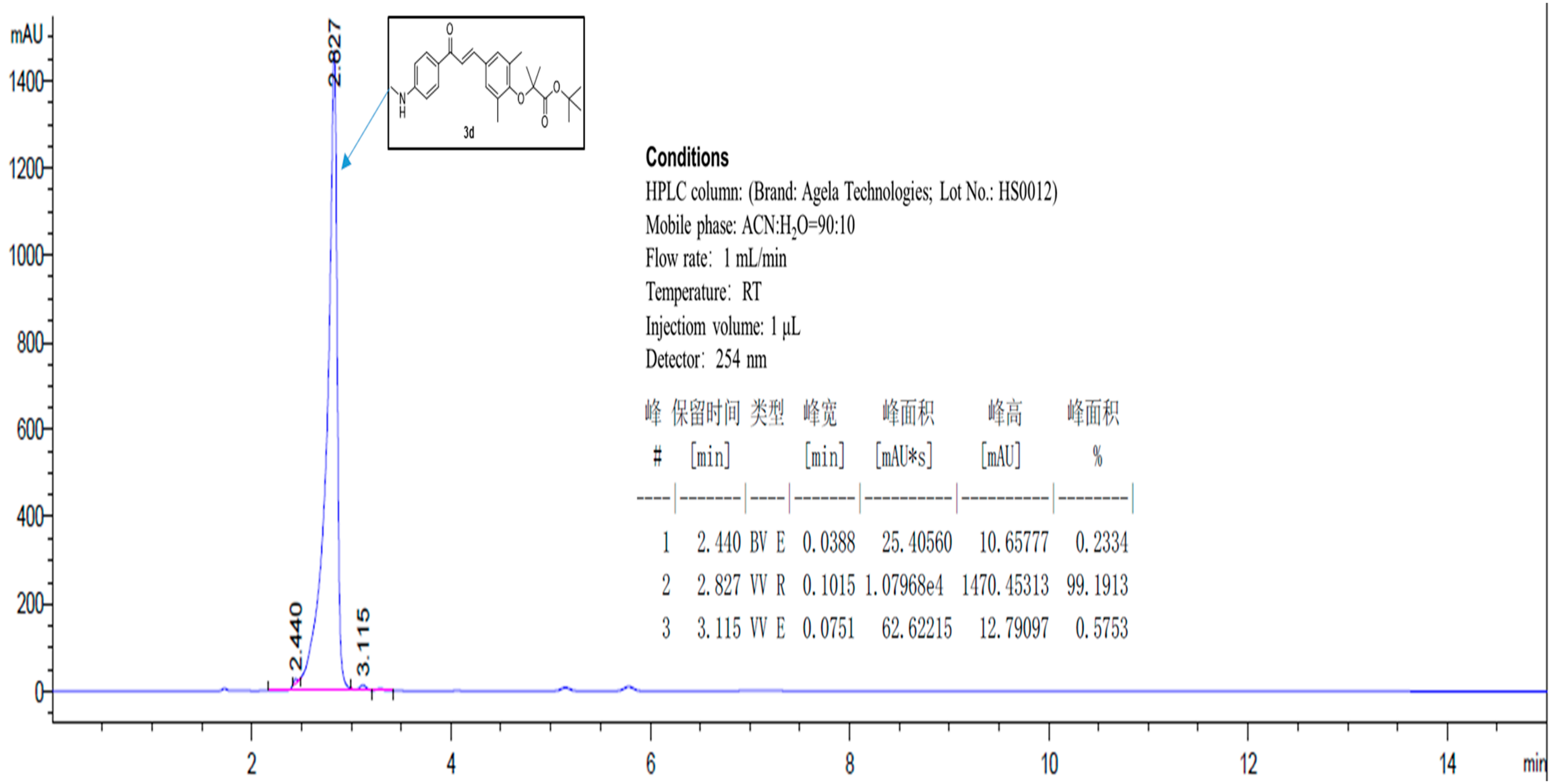

峰 #	保留时间 [min]	类型	峰宽 [min]	峰面积 [mAU*s]	峰高 [mAU]	峰面积 %
1	2.440	BV E	0.0388	25.40560	10.65777	0.2334
2	2.827	VV R	0.1015	1.07968e4	1470.45313	99.1913
3	3.115	VV E	0.0751	62.62215	12.79097	0.5753

Figure S3. HPLC spectrum of compound 3d.
